# Frequency of Extensively Drug-Resistant Gram-Negative Pathogens in a Tertiary Care Hospital in Pakistan

**DOI:** 10.7759/cureus.11914

**Published:** 2020-12-05

**Authors:** Sundas Abbas, Asad Ullah Sabir, Noor Khalid, Sarah Sabir, Sana Khalid, Shawal Haseeb, Muhammad Numair Khan, Waqas M Ajmal, Faryal Azhar, M.Talha Saeed

**Affiliations:** 1 Pathology, Rawalpindi Medical University, Rawalpindi, PAK; 2 Emergency Medicine, Rawalpindi Medical University, Rawalpindi, PAK; 3 General Surgery, Holy Family Hospital, Rawalpindi, PAK; 4 General Surgery, Rawalpindi Medical University, Rawalpindi, PAK

**Keywords:** antimicrobial drug resistance, gram-negative bacteria, extensively drug resistant

## Abstract

Background

Gram-negative bacteria are frequently involved in nosocomial infections. These bacteria have a particular tendency to develop antibiotic resistance and may become extensively drug-resistant (XDR). This study aimed to detect the prevalence of XDR Gram-negative bacteria in a tertiary care hospital in Pakistan.

Materials and methods

Clinical samples were obtained from patients admitted to different inpatient wards and sent for microbial analysis and culture. Antibiotic susceptibility testing of isolates was performed by the disk diffusion method to detect XDR strains.

Results

Antibiotic susceptibility patterns of a total of 673 clinical samples were studied. Of all bacterial isolates, 64% were extensively drug-resistant. *Klebsiella pneumoniae* had the highest percentage of XDR isolates (68.4%), followed by *Pseudomonas aeruginosa* (67.6%) and *Escherichia coli* (56.1%). Most XDR pathogens were isolated from the burn unit (87.7%), followed by the intensive care unit (69.2%) and surgical unit (68.9%).

Conclusions

The rate of extensive drug-resistance is alarmingly high, which calls for strict surveillance and control measures to prevent the development of further resistance. Proper sanitation and rational prescription of antibiotics should be ensured.

## Introduction

Gram-negative bacteria and its subgroup Enterobacteriaceae constitute the normal intestinal flora in most human beings. These bacteria are mainly involved in nosocomial infections and have a particular propensity to develop antibiotic drug resistance [1]. This phenomenon has rendered treatment of these infections challenging, often resulting in increased morbidity and costs of healthcare. Research has identified various mechanisms by which Gram-negative bacteria develop resistance. These include extended-spectrum β-lactamases (ESBLs), carbapenemases, and other antimicrobial resistance genes (ARGs) that are circulated among bacteria via horizontal gene transfer or acquired through mobile genetic elements such as plasmids, integrons, and transposons [2].

Based on the patterns of antibiotic resistance, bacteria can be classified as multidrug-resistant (MDR), extremely drug-resistant (XDR), or pan drug-resistant (PDR). The Center for Disease Control & Prevention (CDC) and the European Center for Disease Control (ECDC) have developed standardized definitions of all these entities, which are now universally accepted [3]. According to these definitions, XDR bacteria are resistant to all but two or fewer antimicrobial categories. In other words, bacteria that remain susceptible to only one or two classes of antimicrobial drugs are extensively drug-resistant [3].

Extensively drug-resistant bacteria are a significant healthcare concern, with their incidence increasing worldwide. The World Health Organization has categorized ESBL-producing Enterobacteriaceae (XDR Enterobacteriaceae) and carbapenem-resistant *Pseudomonas aeruginosa* as ‘critical’ threats and has called for urgent development of new and effective antibiotic treatments against these pathogens [4]. XDR Gram-negative pathogens pose a serious threat to the economy of developing countries like Pakistan, where rates of antibiotic resistance are much higher due to the excessive and non-judicial use of antibiotics [5].

Gram-negative bacilli are implicated in the majority of healthcare-associated infections (HAI) in our setting. Various studies have been done in the past to report resistance patterns, and reports from all over the country have provided evidence of increasing resistance of Gram-negative bacteria in Pakistan [6,7]. Strict surveillance of antibiotic resistance is necessary to be able to take timely measures and devise control strategies for the control of this significant health issue. Such surveillance is also essential to improve patient care, by influencing the choice of empirical antibiotics used as treatment or prophylaxis of infections [8]. Consequently, our study aims to recognize and appreciate the popularity of XDR pathogens in a tertiary care facility in Pakistan and to shed light on the magnitude of the radical problem of antimicrobial resistance faced by healthcare professionals. 

## Materials and methods

This cross-sectional study was conducted at Holy Family Hospital, Rawalpindi, Pakistan, from July 2018 to January 2019. The study was approved by the Institutional Review Board of Rawalpindi Medical University (approval number: RSRS-2017-P-042).

Patients admitted to various inpatient wards who had undergone microbiological analysis of their clinical samples as part of their investigations during their hospital stay, were included in the study. Patients were included irrespective of their antibiotic status. Patients with polymicrobial infections or those with cultures positive for Gram-positive bacteria were excluded. Patient sampling was consecutive, and we included patients from the departments of medicine, surgery, gynecology, pediatrics, burn unit, and the intensive care unit (ICU). Samples collected from patients were blood, urine, pus, sputum, catheter tips, swab sticks, surgical, and burn wounds. These samples were sent to the microbiology laboratory of the hospital for microbial culture and sensitivity.

In the laboratory, specimens were cultured on suitable culture media, which include (but are not limited to) blood agar, MacConkey agar, and chocolate agar. After the identification of bacterial isolates, antibiotic sensitivity tests were performed by the disk diffusion method according to the Clinical Laboratory and Standard Institute criteria [9]. Bacteria were grown on Mueller Hinton agar, and their growth observed around antibiotic disks. Antibiotics used as the first-line for Gram-negative bacteria were ampicillin, gentamicin, cefazolin, tobramycin, co-trimoxazole, ceftazidime, and nitrofurantoin. Ampicillin-sulbactam, amoxicillin-clavulanate, amikacin, piperacillin-tazobactam, cefotaxime, cefepime, moxifloxacin, ciprofloxacin, imipenem, meropenem were used as second-line drugs. Aztreonam, tetracycline, and chloramphenicol were third-line drugs.

Extensively drug-resistant bacteria were defined according to the criteria described by the CDC [3]. Data were statistically analyzed using Statistical Package for Social Sciences (SPSS) version 22.0 for Windows (IBM Corp., Armonk, NY, USA). Frequencies and percentages of XDR bacteria were determined for all clinical isolates.

## Results

A total of 673 isolates were included in this study. The general characteristics of the obtained samples including the patient sex, department of admission, and sample site are given in Table 1. 

**Table 1:** Basic characteristics of all clinical samples in the study (n = 673)

Sample Characteristics	Frequency	Percentage
Patient sex		
Male	366	54.4%
Female	307	45.6%
Department		
Surgery	280	41.6%
Medicine	197	29.3%
Intensive care unit	104	15.5%
Burn center	73	10.8%
Gynecology and obstetrics	11	1.6%
Pediatrics	8	1.2%
Infectious sample site		
Infected wound	241	35.8%
Pus	154	22.9%
Catheter	94	14.0%
Urine	80	11.9%
Sputum	25	3.7%
Blood	15	2.2%
Others	64	9.5%

Among all clinical samples,* P. aeruginosa* was the most common Gram-negative bacterium to be isolated followed by *Escherichia coli* and *Klebsiella pneumoniae*. This is represented in Table 2.

**Table 2:** Frequency of various Gram-negative bacteria isolated (n = 673)

Organism Isolated	Total Frequency	
	Frequency	Percentage
Pseudomonas aeruginosa	257	38.2%
Klebsiella pneumoniae	196	29.1%
Escherichia coli	220	32.7%

**Figure 1:**
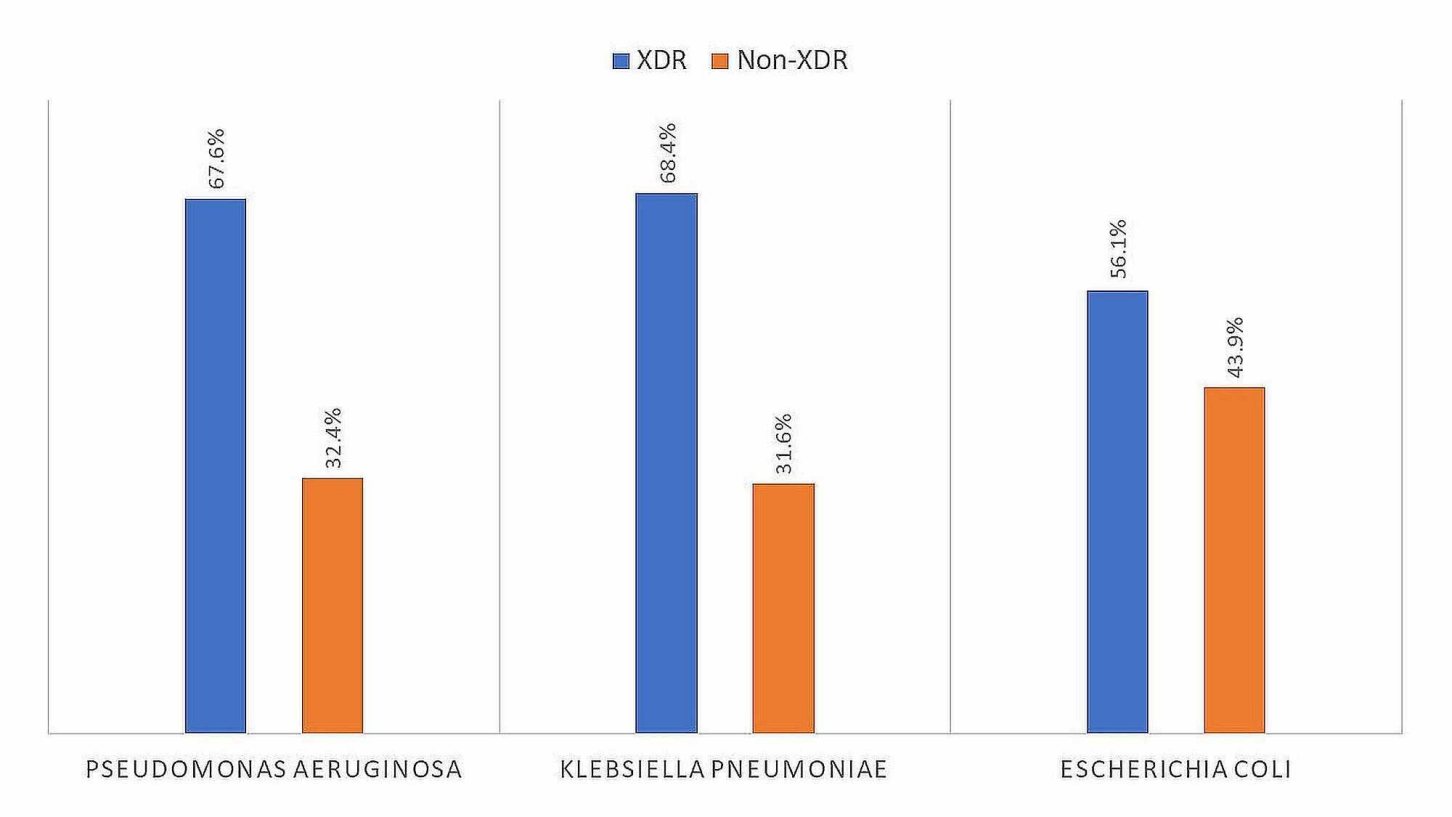
Proportion of XDR among various Gram-negative bacteria XDR: extensively drug-resistant

Out of all 673 bacterial isolates, 431 (64.0%) were extensively drug-resistant. The rest 242 isolates were non-XDR (36.0%). Among the samples studied, *K. pneumoniae* had the highest rate of extensive drug resistance, with 68.4% (n = 134) of its isolates being XDR. It was followed by *P. aeruginosa* with 67.6% (n = 173) XDR isolates, and by *E. coli* with 56.1% (n = 124) of its isolates being extensively drug-resistant (Figure 1).

**Figure 2:**
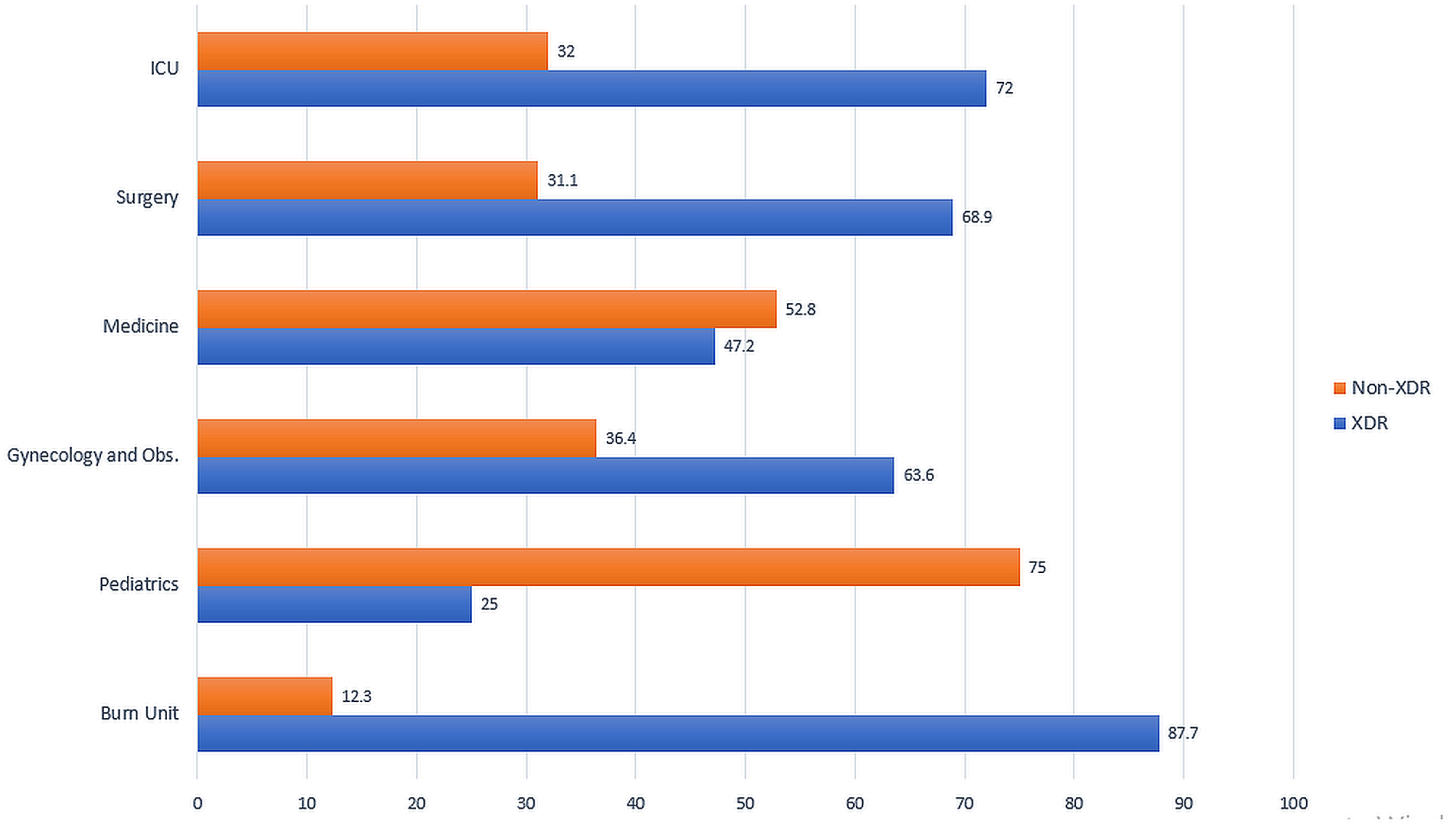
Proportion of Gram-negative XDR bacteria isolated from different specialties (in %) XDR: extensively drug-resistant, ICU: intensive care unit, Obs: obstetrics

Figure 2 shows the proportions of Gram-negative XDR strains isolated from different clinical specialties. The highest percentage of XDR Gram-negative pathogens were isolated from the burn unit (n = 64, 87.7%), followed by ICU (n = 72, 69.2%) and the surgical unit (n = 193, 68.9%). 47.2% (n = 93) of isolates from the medical unit were XDR positive. From the department of gynecology and pediatrics, the rate of XDR Gram-negative bacteria was 63.6 and 25 percent, respectively.

**Figure 3:**
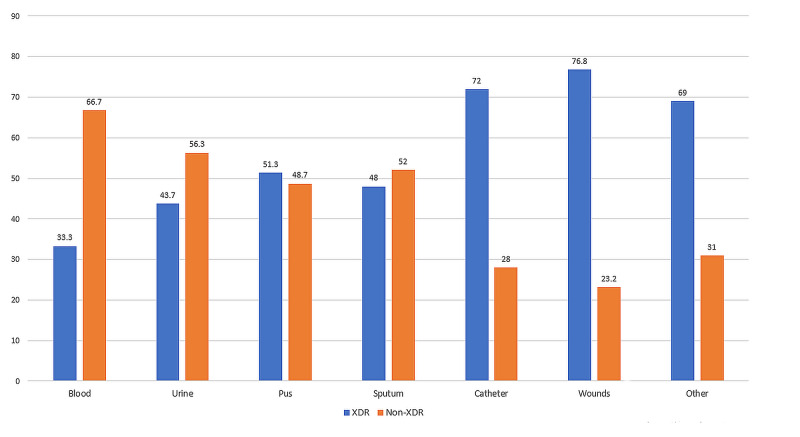
Percentages of Gram-negative XDR pathogens among various isolation sites XDR: extensively drug-resistant

The frequency of isolation of XDR Gram-negative bacteria from clinical isolates of various tissue sites was determined (Figure 3). Of all sites, wound infections were observed to have the highest rate of XDR pathogens (n = 185, 76.8%). This was followed by catheter infections, which were caused by extensively drug-resistant pathogens in 76.6% of the cases (n = 72). 

Eighty percent isolates of *P. aeruginosa* obtained from catheters, and 79.4% of isolates from wound sites were extensively drug-resistant. Similarly, 79.6% isolates of *K. pneumoniae* obtained from catheter sites were XDR. For *E. coli,* the highest percentage of XDR isolates was obtained from non-conventional sites (72.2%). This is shown in Table 3.

**Table 3:** Incidence of XDR Gram-negative pathogens in various isolation sites XDR: extensively drug-resistant

Site	Organism								
	Eschericia coli			Klebsiella pneumoniae			Pseudomonas aeruginosa		
	XDR	Non-XDR	XDR %	XDR	Non-XDR	XDR %	XDR	Non-XDR	XDR %
Blood	1	0	100%	2	2	50.0%	1	9	10.0%
Urine	23	35	39.7%	6	8	42.9%	6	2	75.0%
Pus	27	29	48.2%	17	13	56.7%	35	33	51.5%
Sputum	5	4	55.6%	5	7	41.7%	2	2	50.0%
Catheter	21	9	70.0%	39	10	79.6%	12	3	80.0%
Wounds	34	15	69.4%	51	15	77.3%	100	26	79.4%
Others	13	5	72.2%	14	7	66.7%	17	8	68.0%

## Discussion

The phenomenon of antimicrobial drug resistance is growing at an alarming rate, with the frequency of MDR and XDR bacteria increasing daily. In our study, we found that 64% of Gram-negative bacteria isolated from hospital settings were extensively drug-resistant (XDR). *K. pneumoniae* was the most frequently resistant pathogen, with 68.4% of its isolates being XDR. The highest percentage of XDR Gram-negative pathogens was from the burn unit (87.7%), and catheters and wound infections were the most common sites of XDR bacteria.

The results of our study are remarkable in many aspects. Firstly, we found the percentage of extensive drug resistance to be much higher than other studies in the past. In a study from Eastern India, the XDR Gram-negative load was found to be 41.3% [10]. Another study from 2016, that reported the prevalence of XDR organisms among tracheal aspirates obtained from an ICU of a tertiary care hospital in Pakistan, showed that 56.5% of isolates belonged to the XDR category [11]. In contrast, our study found the rate of XDR among Gram-negative bacteria to be 64%.

The higher percentage of XDR bacteria found in our study might be reflective of the increasing resistance among Gram-negative pathogens, especially in developing countries [12]. Moreover, poor infection control and sanitization in tertiary care public hospitals allow for the rapid and unchecked transmission of drug-resistant pathogens. Besides, there is an unrestrained availability of antibiotics in local markets. A trend of unchecked and needless prescription of antibiotics exists in the hospital setting. All of these factors contribute to the increased drug resistance rates, which are reflected in our study results [12].

Secondly, our study found the frequency of XDR pathogens among various bacterial species to be the highest in* K. pneumoniae*, followed by *P. aeruginosa* and *E. coli*. These findings are different from previous studies that have reported *P. aeruginosa* to be the most resistant Gram-negative pathogen [13]. Our findings are explained and supported by recent studies from Asia which have highlighted increasing antibiotic resistance among *K. pneumoniae* isolates in this region. In a study conducted in Iran in the year 2020, researchers observed that antibiotic resistance in *K. pneumoniae* was much higher than studies conducted across other regions of the world [14]. Other recent studies have also expressed concern over the growing resistance pattern of Klebsiella in the Asian region, and it is feared that, if unchecked, XDR* K. pneumoniae* might emerge as a huge future problem in this region [15].

Among different hospital specialties, the highest rate of extensive drug resistance was present in the burn unit. Of all isolates from the burn unit, 87.7% were XDR. This finding is in accordance with a recent study conducted among burn patients in Iran, which evaluated antibiotic resistance patterns among *P. aeruginosa* isolates in burn patients. They found 87.5% of isolates to be extremely drug-resistant, which is similar to our findings [16]. Another study conducted in 2019 across burn patients in India reported the frequency of extensive drug resistance among Pseudomonas isolates as 71.25%. The frequencies of XDR Klebsiella and XDR *E. coli* were 68.63% and 58.33%, respectively [17]. Although the proportion of XDR pathogens in our study was higher than that reported in India, the general pattern of increased drug resistance among bacteria in burn wounds is comparable.

It might also be worthwhile to note that the percentage of XDR bacteria observed in intensive care units in our hospital is much more than that reported in other studies. We found that 69.2% of isolates of Gram-negative Enterobacteriaceae from the ICU were extensively drug-resistant. These numbers are significantly higher than those reported by a study conducted in Nepal in 2017, in which the rate of XDR Gram-negative bacteria in the intensive care unit was 43.3% [18]. ICUs have traditionally been the hub of the development and spread of antibiotic-resistant bacteria [19]. Most countries, however, have now adopted strict control measures, including antibiotic stewardship programs and sanitation measures, which have resulted in the control of the resistance rate. Such programs are lacking in our setup and the results of our study call for immediate action.

Public hospitals in developing countries serve to provide both primary and tertiary level healthcare facilities to a large proportion of their population. The growing rate of antibiotic resistance among organisms isolated in hospital-acquired infections in this setting is alarming, particularly because the exposure of such a large number of people to XDR pathogens will eventually be the source of the spread of these pathogens in the community. Tertiary care hospitals in Pakistan must adopt effective strategies to control the spread of such organisms in the healthcare setting. Both 'reservoir-based' and 'transmission based' strategies ought to be adopted for the control of all resistant pathogens and especially Gram-negative bacteria [20]. 

There are many guidelines and proposed strategies for the control of antibiotic resistance. At the beginning of the century, the CDC proposed basic measures to prevent the development of antibiotic-resistant infections. These included: avoiding infections by hygienic measures, strict surveillance, developing new antibiotics against resistant bacteria, and enlisting administrative support [21]. Based on similar principles, the 2015 French recommendations for the control of the transmission of emerging XDR bacteria call for strict hygiene measures, source control, and screening to prevent outbreaks [22]. The antimicrobial stewardship program (AMS), which has been developed to avoid overuse of antibiotics, especially in the ICU setting, is another effective strategy to help reduce the burden of resistant bacteria [19].

Our personal recommendations for the management of this problem include the development of strict guidelines for antibiotics use by hospital administration and policymakers and ensuring the implementation of these guidelines. Physician awareness programs and quality improvement projects (audits) should be undertaken to ensure compliance by healthcare professionals. All patients should undergo culture and sensitivity testing before antibiotics administration, and the use of antibiotics targetting resistant bacteria should not be used as empirical therapy. It has also been found by recent studies that empirical therapy targetting resistant species has no mortality benefit over the use of standard antibiotics, and only contributes to the phenomenon of increased resistance [23].

There are a couple of limitations to our study. First, a study done in a single healthcare center may not speak for the general prevalence of XDR pathogens in the hospital setting. A multi-center study with a larger cohort and a longer duration of study would be required to achieve generalizable results. Second, we included only three species of Gram-negative bacteria in our study and did not account for other important bacterial species such as *Acinetobacter baumannii*, which is a notable cause of nosocomial infections. Laboratory testing for Acinetobacter is costly, and therefore, not routinely carried out in the majority of laboratories in our countries.

## Conclusions

The percentage of extensively drug-resistant Gram-negative bacteria was found to be 64%, which is alarmingly high. Strict surveillance and control measures should be undertaken to prevent the development of further resistance, which will decrease treatment options to a minimum. Judicious use of antibiotics after sensitivity testing should be practiced in all healthcare centers, and resistance patterns of Gram-negative bacteria should be studied on a cellular and genetic level to develop newer therapeutic options against these pathogens.
